# Classification of Autism Spectrum Disorder Using Random Support Vector Machine Cluster

**DOI:** 10.3389/fgene.2018.00018

**Published:** 2018-02-06

**Authors:** Xia-an Bi, Yang Wang, Qing Shu, Qi Sun, Qian Xu

**Affiliations:** College of Mathematics and Computer Science, Hunan Normal University, Changsha, China

**Keywords:** random support vector machine cluster, neuroimaging, autism spectrum disorder, classification, feature selection

## Abstract

Autism spectrum disorder (ASD) is mainly reflected in the communication and language barriers, difficulties in social communication, and it is a kind of neurological developmental disorder. Most researches have used the machine learning method to classify patients and normal controls, among which support vector machines (SVM) are widely employed. But the classification accuracy of SVM is usually low, due to the usage of a single SVM as classifier. Thus, we used multiple SVMs to classify ASD patients and typical controls (TC). Resting-state functional magnetic resonance imaging (fMRI) data of 46 TC and 61 ASD patients were obtained from the Autism Brain Imaging Data Exchange (ABIDE) database. Only 84 of 107 subjects are utilized in experiments because the translation or rotation of 7 TC and 16 ASD patients has surpassed ±2 mm or ±2°. Then the random SVM cluster was proposed to distinguish TC and ASD. The results show that this method has an excellent classification performance based on all the features. Furthermore, the accuracy based on the optimal feature set could reach to 96.15%. Abnormal brain regions could also be found, such as inferior frontal gyrus (IFG) (orbital and opercula part), hippocampus, and precuneus. It is indicated that the method of random SVM cluster may apply to the auxiliary diagnosis of ASD.

## Introduction

Autism spectrum disorder (ASD) is mainly reflected in the communication and language barriers, difficulties in social communication, and it is a kind of neurological developmental disorder (Karten and Hirsch, 2015; Khundrakpam et al., 2017). The behavioral phenotype of ASD is well-depicted but its etiology and pathogenesis is rarely known (Amaral et al., 2008). According to the results of Hallmayer et al. (2011), the causes of ASD mainly include genetic and environmental risk factors. Some of the symptoms of ASD generally appear in about 2 years old (Ecker et al., 2015), thus the early diagnosis is needed (Plitt et al., 2015). It is recognized that traditional clinical methods cannot well distinguish patients from healthy controls (HC) (Mwangi et al., 2012). And it may not be complex enough to capture the abnormal brain regions in individuals who suffering from ASD (Uddin et al., 2011). To avoid this disadvantage, machine learning is introduced in the neuroimaging field. It is a valid means to extract messages from neuroimaging data and further predict the future changes of the disease (Klöppel et al., 2012; Orrù et al., 2012). Among numerous machine learning methods, support vector machines (SVM) is an excellent classification method (Zhang et al., 2015). SVM has distinct merits such as the higher classification accuracy (Zhang and Wu, 2012) and no need for a large number of training samples to avoid over-fitting (Li et al., 2010). Thus, SVM has aroused widespread concern of researchers in the field of neuroimaging (Sundermann et al., 2014).

In the researches of machine learning, SVM has been applied to classify ASD from corresponding controls. Gori et al. (2015) extracted features from the gray matter subregions, then these features were used in SVM to identity ASD from HC and the area under ROC curve (AUC) is 0.74. Jin et al. (2015) proposed an original multi-kernel SVM classification method to classify ASD from HC and the accuracy can reach to 76%. Chen et al. (2016) used the SVM to classify 112 adolescent subjects with ASD and 128 HC, and the classification accuracy was 79.17%. Odriozola et al. (2015) used the SVM based on functional magnetic resonance imaging (fMRI) data to classify 20 children with ASD and 20 typically developing (TD) peers, and the result showed 85% classification accuracy. Chanel et al. (2016) used the method of SVM and Recursive Feature Elimination (RFE) based on fMRI data to classify ASD from HC, and the result showed good classification accuracy (up to 92.3%).

These SVM classification studies on ASD have achieved relatively high classification accuracy in the range of 70–93% compared with traditional methods. They usually employed a single SVM and common features such as functional connections, gray matter volume to classify ASD from HC, and the classification accuracy is generally lower than 90%. In this paper, a novel method of random SVM cluster is proposed and several graph metrics of brain functional connectivity (e.g., local efficiency, shortest path) are employed to classify ASD and typical controls (TC). This method has some good performance. Firstly, the classification accuracy reaches to a higher level based on all the features. Then, we could find out the optimal feature set, the classification accuracy could also reach to the same level based on the optimal feature set. Thirdly, on the basis of the optimal feature set, we could find out the abnormal brain regions such as inferior frontal gyrus (IFG) (orbital and opercula part), hippocampus, and precuneus. Thus, the random SVM cluster may apply to the auxiliary diagnosis of ASD.

## Materials and methods

### Demographic information

The Autism Brain Imaging Data Exchange (ABIDE) database (http://fcon_1000.projects.nitrc.org/indi/abide/) (Di Martino et al., 2014) contains a variety of neuroimaging data. And the resting-state fMRI data include 539 ASD and 573 age-matched TC. There are 12 kinds of image protocols. This study chooses one of the image protocols, details as follows. MRI scanner = 3.0-T Siemens, TR = 3,000 ms, TE = 28 ms, data matrix = 64^*^64, Pixel Spacing X = 3.0 mm, Pixel Spacing Y = 3.0 mm, Flip Angle = 90°, Slice Thickness = 0.0 mm, no slice gap, axial slices = 34, time points = 120. Finally, 61 subjects with ASD and 46 TC met the image protocol. The original studies included in ABIDE received approval from each site's Institutional Review Board (IRB). All images were obtained with informed consent according to procedures established by human subject research boards.

Only 84 of 107 subjects are utilized in experiments because the translation or the rotation of 7 TC and 16 ASD patients has surpassed ± 2 mm or ± 2°. Table 1 shows the basic information of 84 participants. To assess the gender and age discrepancies between ASD group and TC group, we employed chi-square test and two-sample *t-*test respectively. The results show no considerable discrepancies between TC group and ASD group in gender (as the *p*-value is 0.359 and >0.05) and age (as the *p*-value is 0.278 and >0.05).

**Table 1:** Basic information of ASD and TC.

**Variables (Mean ± SD)**	**Autism (*n* = 45)**	**TC (*n* = 39)**	***P* value**
Gender (M/F)	41/4	33/6	0.359
Age (years)	13.4 ± 2.4	12.9 ± 1.7	0.278

*ASD, Autism spectrum disorder; TC, typical controls*.

### Data preprocessing

Because of the lower signal-to-noise ratio, all the fMRI images are preprocessed by the Data Processing Assistant for Resting-State fMRI (DPARSF) software (Cui et al., 2015) (http://d.rnet.co/DPABI/DPABI_V2.3_170105.zip). The whole preprocessing procedures include:(1) Converting DICOM to NIFTI, (2) Removing the first 10 time points, (3) Slicing timing, (4) Realigning, (5) Normalizing with the echo-planar imaging (EPI) template, (6) Smoothing, (7) Removing the linear trend, (8) Temporal filtering, (9) Removing covariates.

### Graph theory application

Our brain is consisted of different regions. Although each region carries out its own tasks, they often interconnect with each other, and the connections form the brain network. Graph could be employed to represent networks. A graph has two major components, nodes and links. fMRI image could be divided into 90 regions based on the Automatic Anatomical Labeling (AAL) (Tzourio-Mazoyer et al., 2002) atlas. We make these regions as the nodes of brain network, so there are 90 nodes. An average of the time series of a region's all voxels can be used to capture the mean time series of this region (Khazaee et al., 2016). The average time series of every two brain regions can be employed to calculate the Pearson correlation coefficient which is made as the link of the brain network (Khazaee et al., 2015), so there are 4,005 (90^*^89/2) weighted edges. Then we take the absolute value of the correlation coefficient and set a suitable threshold for the connectivity matrix to get an adjacency matrix. In this paper, the threshold is 0.25. Graph metrics calculated for this paper included: degree, shortest path, local efficiency and clustering coefficient. Thus, results have 90°, 4,005 shortest paths, 90 local efficiency, and 90 clustering coefficients. These graph metrics were the subsequent experimental features.

### The random SVM cluster

#### The design of the random SVM cluster

Previous classification studies generally used a single SVM as classifier. Although sometimes of the studies have good performance, it is not stable and always be affected by many factors such as the form of the kernel, the argument of the kernel, the penalty coefficient. A new classifier based on the random SVM cluster is proposed in this paper. It is featured with universality, stable performance and high accuracy.

The process of the random SVM cluster is as follows. Firstly, a sample set is divided into a training set and a test set. Then partial samples are randomly selected from the training set and partial features are extracted from all the sample features to establish a single SVM. The process is repeated for several times to form a random SVM cluster. When the sample of test set enters into the random SVM cluster, multiple SVMs make decisions at the same time and then we use the majority of votes to determine the category of the sample.

In the above process, the randomness of the random SVM cluster is reflected in two aspects, one is the randomness of sample selection, and another is the randomness of feature selection. Therefore, our model is universal and avoids the influence of many factors.

**Figure 1:**
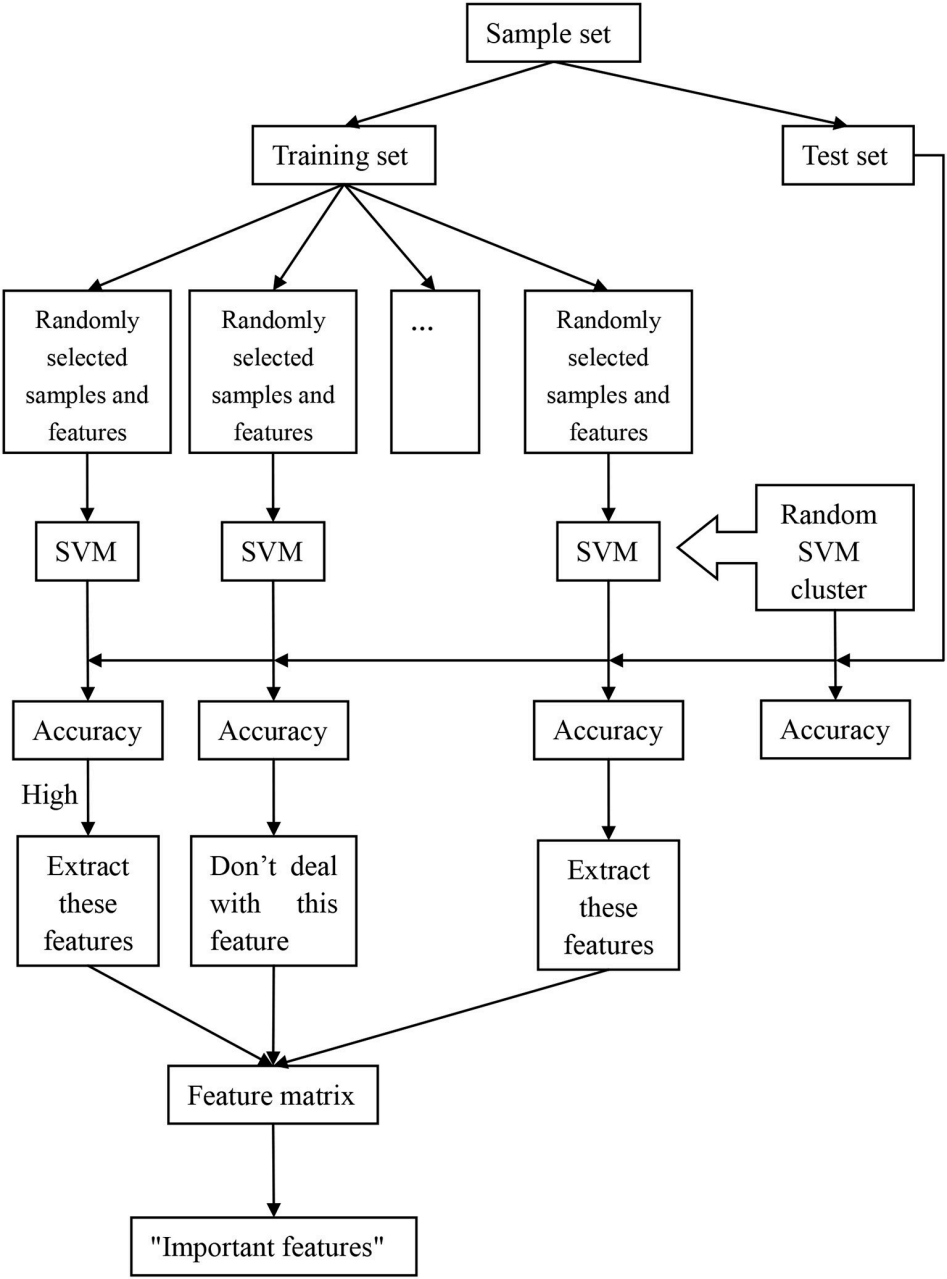
The overall framework of random SVM cluster.

Based on the performance evaluation of a single SVM, the features of the classifier with higher classification accuracy constitute the feature matrix. Then the frequency of each feature in the features matrix is counted. The features with higher frequency is called “important feature.” Figure 1 is an overall flow chart of the random SVM cluster.

#### The classification of the random SVM cluster

The application of the random SVM cluster in fMRI is to construct a random SVM cluster based on the brain functional data of the subjects, and then it is used for classification as well as feature selection.

In this paper, the sample set is ${\left\{\left({\text{x}}_{\text{i}},\;{\text{y}}_{\text{i}}\right)\right\}}_{i}^{84}$, where **x**_**i**_ is the sample feature, *y*_*i*_ is the category label. Each sample has 4,225 (90 + 4,005 + 90 + 90) features and the label of the ASD patient is −1 and the TC is +1.

Firstly, the 84 samples are divided into 58 training samples and 26 test samples based on the ratio of 7:3. Then 50 samples are randomly selected from the 58 training samples and 62 features are randomly selected from 4,225 features, which form a single SVM using Radial Basis Function (RBF) as kernel provided by the SVM toolbox (http://see.xidian.edu.cn/faculty/chzheng/bishe/indexfiles/indexl.htm) (Gunn, 1998). The width argument of RBF equals to 3, and penalty coefficient equals to Inf. The process is repeated for 500 times and these 500 SVMs constitute into a random SVM cluster.

When the 26 test samples enter into the random SVM cluster, and the 500 SVMs in the random SVM cluster make decisions simultaneously. The result of the 500 SVMs is counted, and the label with more votes is recorded as the predictive label of the sample. Thus, the predictive label of 26 test samples can be obtained. The number of samples with the same predictive label and the real label is divided by 26, which is the classification accuracy of the random SVM cluster.

We set up a random SVM cluster with 500 SVMs in the above, but we not sure whether the number of 500 is suitable. Therefore, it is necessary to find the optimal number of SVMs to set up the random SVM cluster. In this paper, we take the accuracy of the random SVM cluster as the criterion. The number of SVM in the random SVM cluster with the highest accuracy is the optimal SVM number.

#### Extracting features from random SVM cluster

In the random SVM cluster, the performance of each SVM is different because the selected features for each SVM are different. Since the “important features” make a significant contribution to the classification performance of a single SVM, we could find the “important features” through the SVM with higher classification accuracy. The specific approach is as follows.

First of all, a random SVM cluster is built. Then the 26 test samples are used to test the classification of each SVM performance. The features of the first 100 SVMs with better classification performance constitute the feature matrix. The first 400 features with the higher frequency are referred as the “important features.”

We randomly select 62 features from the first *q*(70 ≤ *q* ≤ 400) features in the “important features” to build a random SVM cluster. The accuracy of the random SVM cluster is used as the criterion. The first *q* features that corresponding to the random SVM cluster with the highest accuracy are the optimal feature set. Those *q* features are the result of feature selection with random SVM cluster and can be seen as distinguishing features between the ASD patient and TC.

This paper uses the optimal feature set to find the abnormal brain regions. To show the abnormal brain region, the key is to find the weight of each brain region. Firstly, we find the features associated with certain region from the optimal feature set. Then the number of these features is made as the weight of the region.

## Results

### The performance of a random SVM cluster

**Figure 2:**
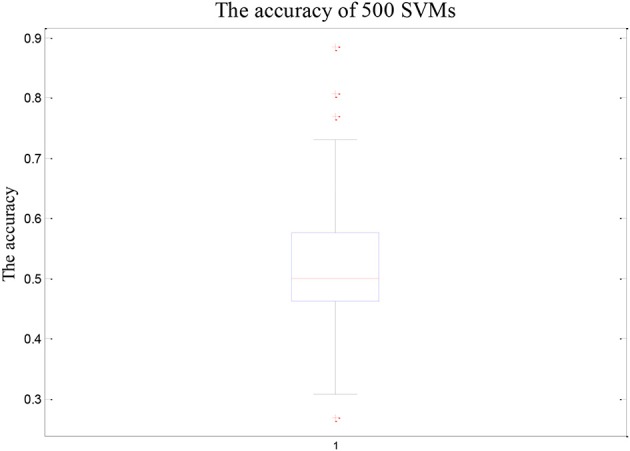
The accuracy of 500 SVMs.

As shown in Figure 2, the overall accuracy of the 500 SVM is lower. On the contrary, the accuracy of the random SVM cluster is as high as 96.15%. This is sufficient to show that the performance of the random SVM cluster is much better than a single SVM.

### The optimal number of base classifiers

**Figure 3:**
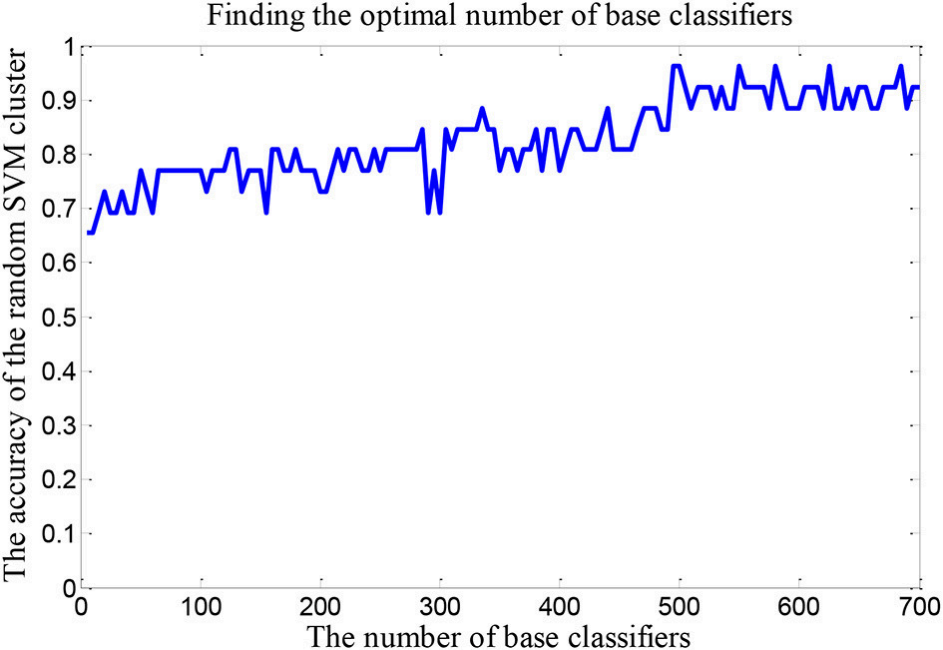
The optimal number of base classifiers.

The number of SVMs in the corresponding random SVM cluster with the highest classification accuracy is the optimal number of classifiers. First, we change the number of base classifiers from 5 to 700 and the step is 5. Then we count the classification accuracy of random SVM cluster with different number of classifiers. Finally, we sort the results and draw the corresponding graph as shown in Figure 3. The accuracy of the random SVM cluster reached a maximum of 96.15% and stabilized when the number of SVM is 500. Thus, 500 is regarded as the optimal number of base classifiers. In the subsequent experiments, we also use 500 as the number of classifiers.

### Retention of the “important features”

The important features should meet the following two criterions: (1) The single SVM corresponding to these features has high classification accuracy. (2) These features have high frequency.

First of all, this study sorts the accuracy and picks out the features of first 100 SVMs to form a 100^*^62 matrix. Then we count the frequency of each feature number in this matrix. The highest frequency is 7 and the corresponding features are the shortest path between PreCG.R and IFGtriang.R, IFGoperc.R and PHG.L, REC.L and SMG.L, ORBsupmed.R and TPOmid.R. Table 2 lists the features whose frequency is 6 and 7. Since these features are all the shortest paths between two brain regions, only two brain regions corresponding to the shortest paths are listed in the Table 2.

**Table 2:** The part of features with higher frequency.

**Frequency**	**Feature**
7	PreCG.R-IFGtriang.R, IFGoperc.R-PHG.L, REC.L-SMG.L, ORBsupmed.R-TPOmid.R
6	OLF.R-HIP.R, INS.L-HIP.R, ORBinf.R-PHG.L, SFGdor.R-LING.L, ORBsup.L-FFG.L, REC.L-SPG.R, ROL.L-SMG.L, SMG.R-PCL.L, HIP.L-CAU.R, ORBsupmed.L-PUT.L, REC.R-HES.L, PCUN.R-MTG.R IFGoperc.R-STG.L, SMA.R-STG.L, ORBsup.L-STG.R

### The optimal feature set

**Figure 4:**
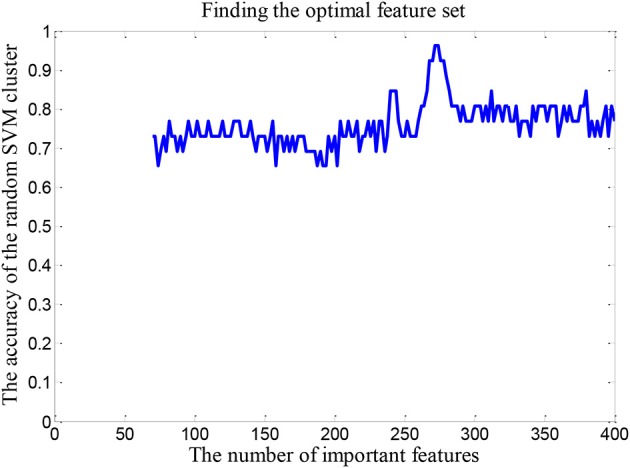
The number of optimal feature sets.

In order to find the optimal feature set, it is necessary to change the rule of the selected features. Firstly, we retain the first 70 dimensional features in the “important features” as the total dimension. Then the 62 dimensional features were randomly selected from the 70 dimensional features to build a random SVM cluster. Next, we change the number of the total dimension. The number of the total dimension is from 70 to 400 and the step is 2. Then we calculate the classification accuracy of random SVM cluster with different number of total dimension. Finally, we sort the results and draw the corresponding graph as shown in Figure 4. The accuracy of the random SVM cluster reached a maximum of 96.15% and stabilized when the first features number is 272. Therefore, the optimal feature set is composed by the first 272 features.

### The abnormal brain regions

**Figure 5:**
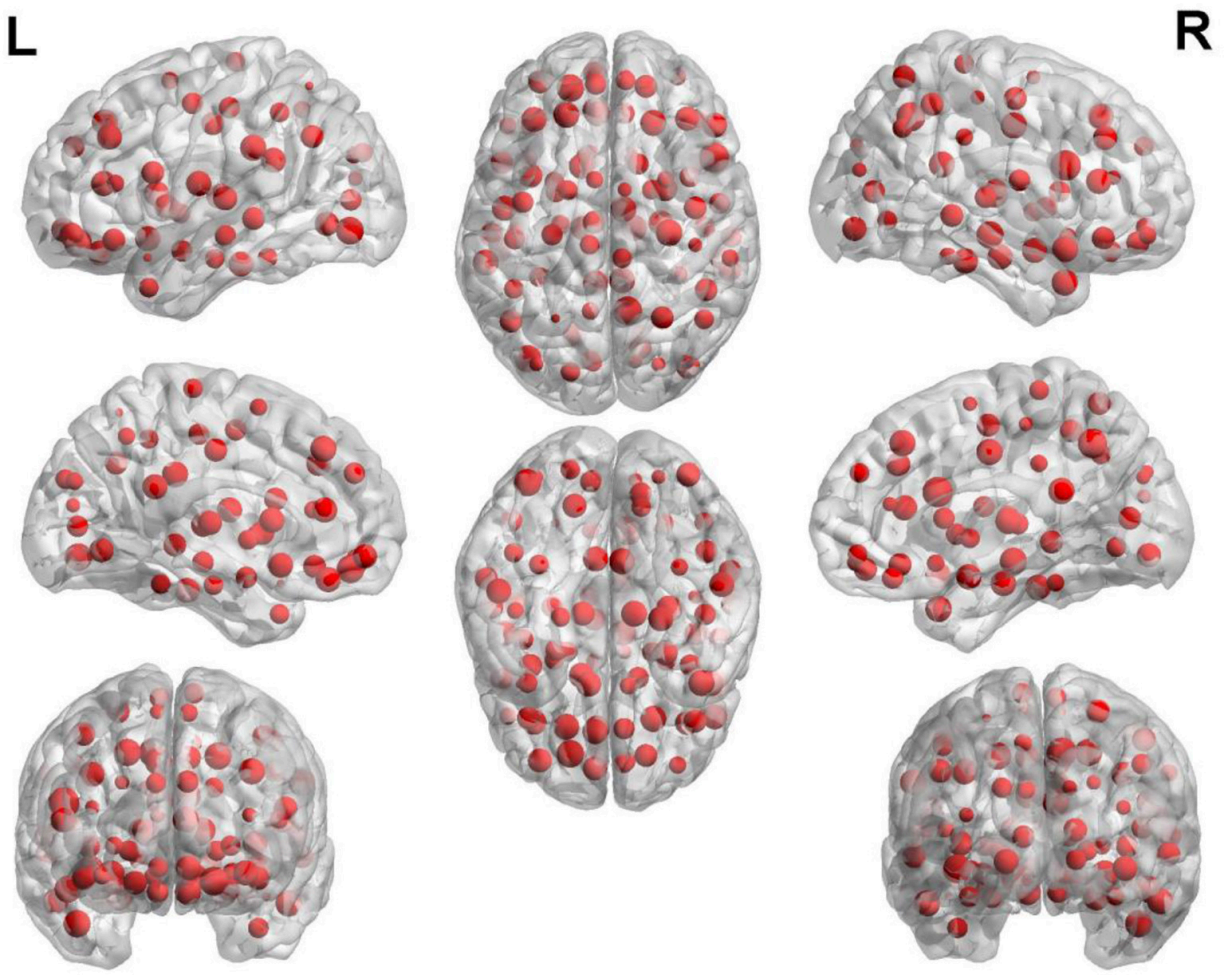
The weight of each brain region.

The weight of each brain region is displayed in Figure 5. In this figure, the point represents the brain region. The brain regions with higher weight are shown in Table 3. The regions with the greater weight are listed as follows: the right IFG (opercular part), the right precuneus, superior frontal gyrus (orbital part), the left inferior occipital gyrus, the right hippocampus, the bilateral superior frontal gyrus (dorsolateral), the right median cingulate and paracingulate gyri, the right posterior cingulate gyrus, the left supramarginal gyrus, the right thalamus, the right superior, and middle temporal gyrus.

**Table 3:** The brain regions with higher weight.

**Weight**	**Region**
15	IFGoperc.R
13	PCUN.R
11	ORBsup.L IOG.L
10	HIP.R
9	SFGdor.L SFGdor.R DCG.R PCG.R SMG.L THA.R TPOsup.R TPOmid.R

## Discussion

### Classification effect

In the experiment, we choose the specific values of some parameters of the random SVM cluster and now we discuss the parameter setting. On one hand, we make the RBF as kernel of a single SVM, the width argument of RBF equals to 3 and penalty coefficient equals to Inf. Although these parameter values are selected in this paper, we have tried other parameter values in the experiment and there is no significant difference in the performance of random SVM cluster. This shows that the random SVM cluster is universal. On the other hand, we make 0.25 as the threshold of the function connection network. Then we calculate the four graph metrics of the network. The larger the threshold is, the smaller the degree is and the larger the shortest path is. The usage of these graph metrics makes the accuracy of random SVM cluster lower. In turn, the smaller the threshold is, the greater the degree is and the smaller the shortest path is. Similarly, the usage of these graph metrics makes the accuracy of random SVM cluster lower. We found that the optimal threshold was 0.25 by dozens of experiments.

In recent years, there are various studies on the classification of ASD. For instance, Chen et al. (2015) used the SVM in combination with a fresh feature selection algorithm to classify 126 participants with ASD and 126 TD, and the classification accuracy was <70%. Jin et al. (2015) proposed an original multi-kernel SVM classification method to classify ASD from HC and the accuracy can reach to 76%. Anderson et al. (2011) used a leave-one-out classifier to discriminate 40 ASD from 40 controls with 79% total accuracy. Chen et al. (2016) used the SVM to classify 112 adolescent subjects with ASD and 128 HC, and the classification accuracy was 79.17%.

The majority of the classifier's accuracy is not higher than 90%. We employ a random SVM cluster to identify ASD and TC with the accuracy of 96.15%. Furthermore, the accuracy of the random SVM cluster reached a maximum of 96.15% and stabilized from 500 SVMs. It is sufficient shows that our method possesses an unexceptionable classification performance.

In this paper, we first establish a random SVM cluster, and then find the optimal number of classifiers based on the accuracy of the random SVM cluster. Then the important features are retained on the basis of the optimal number of the base classifiers. According to the accuracy of the random SVM cluster, the optimal feature set could also be discovered. Finally, the number of selected features is 272. These features constitute the optimal feature set for the random SVM cluster to distinguish ASD patients and TC, and the accuracy could also reach to 96.15%.

### Analysis of the brain regions with greater weight

The experimental results show that the IFG, precuneus hippocampus, and cingulated cortex are the mainly abnormal regions of ASD. The following are the analyses of these regions in detail.

#### Inferior frontal gyrus (IFG)

The right IFG possesses the largest weight in the experimental results which implied that this region is an indispensable part in the classification of our method.

Inferior frontal gyrus, bilateral amygdala, and hippocampus are associated with facial emotion recognition (Ji et al., 2016). During target-oriented actions or when observing the same exercise behavior, the IFG is active (Hamzei et al., 2015). The IFG is a part region of lateral prefrontal cortex which affects regulating mood and attention (Sagaspe et al., 2011; Ochsner et al., 2012; Vanderhasselt et al., 2012). In healthy individuals, the responsiveness of IFG to terrible faces was positively related to the inhibition of amygdala responses, but was negatively related to trait anxiety (Mujica-Parodi et al., 2009). Doricchi et al. (2010) and Shulman et al. (2009) found that the right IFG was activated only when redirecting to an unexpected stimulus.

The abnormal IFG was found in several ASD studies. Keehn et al. (2016) found that compared with the TD group, the activation of the right IFG in the autism group increased obviously. In the study of Grezes et al. (2009) and Philip et al. (2010), the authors found that activation of the right IFG and inferior temporal gyrus in ASD participants decreased when doing the task of fearful gestures. Kim et al. (2015) found that ASD individuals appear to have lower activation in the right IFG than typically developing children (TDC) when fearful face were given to them. Gaffrey et al. (2007) found that less activation of left IFG in the ASD patients than controls.

The abnormal IFG probably bring about impairment in ASD patients such as facial emotion recognition impairment, attention impairment. The experimental result contributes to the clinical diagnosis and treatment of ASD.

#### Precuneus (PC)

The precuneus possesses the relatively larger weight in the experimental results which shows that the precuneus is a considerable part in the classification of our method.

Precuneus is a subregion of superior parietal cortex which is connected with consciousness and self-processing (Cavanna and Trimble, 2006). According to the previous studies, active precuneus is related to the degree of autocorrelation of the retrieved judgments (Lou et al., 2004), and the connectivity of the precuneus is related to the degree of consciousness of a person (Vanhaudenhuyse et al., 2009). It is observed that the precuneus is not activated at any stage of sleep (Maquet et al., 1997) or in vegetative states (Crone et al., 2011). Francis et al. (2016) observed that reduced activation in the precuneus when doing the recognition task. Ashizuka et al. (2015) disclosed that the precuneus is selectively activated during the polite judgment task. From the fMRI studies, we can see that the precuneus participated in a lot of highly integrated duty, including spatial guidance behavior, visual spatial images, and awareness (Mailo and Tang-Wai, 2015).

The abnormal precuneus was found in the abundant studies of ASD. For example, Schulte-Ruther et al. (2011) mentioned that the precuneus activation was positively related to compassion in ASD subjects rather than control subjects. Aoki et al. (2015) found hyperactivation in the cortical structures of ASD patients, including right precuneus and bilateral thalamus. Bookheimer et al. (2008) found that the precuneus was the only region in the ASD group which showed a strong activation when performed a post hoc identification. Cheng et al. (2017) found that functional connectivity of precuneus and orbitofrontal reduced significantly in autism. Silani et al. (2008) discovered that ASD patients displayed obviously less activities in precuneus.

The abnormal precuneus is likely to cause the consciousness disorder and poor integration ability in ASD patients. The experimental result may help in the clinical diagnosis and treatment of ASD.

#### Hippocampus

The hippocampus possesses the relatively larger weight in the experimental results which shows that the hippocampus also is a critical part in the classification of our method.

Hippocampus and amygdala are the key brain areas that involved in emotional memory (Mackiewicz et al., 2006). Some studies on fMRI in patients with depression have displayed that hippocampus is overactive when it comes to tasks related to working memory (Harvey et al., 2005; Walsh et al., 2007). The hippocampus is especially responsible for learning, making up fresh memories, and spatial navigation, and belongs to the limbic system (Ding et al., 2015). Dennis and Thompson (2014) found that the functional connectivity of hippocampus existed during the encoding memory task.

The abnormal hippocampus was found in several ASD studies. For instance, Cooper et al. (2017) found that functional connectivity of hippocampus reduced markedly in ASD group. ASD was associated with a raising relative hippocampus volume (Maier et al., 2015). Stanfield et al. (2008) found that the volume of hippocampus and amygdala was abnormal in ASD. Via et al. (2011) discovered that there was obviously decreased gray matter volume of bilateral amygdala-hippocampus complex in ASD patients. Yu et al. (2011) discovered that gray matter volume of hippocampus decreased distinctly in participants with ASD.

The abnormal hippocampus may result in memory and learning impairment in ASD patients. The experimental result may boost the clinical diagnosis and treatment of ASD.

#### Cingulate gyrus

The cingulate gyrus possesses the comparatively larger weight in the experimental results which shows that cingulate gyrus is a decisive part in the classification of our method.

Numerous studies have pointed out that anterior cingulate cortex (ACC) affects basic cognitive processes, including motivation, making decision, learning, and monitoring errors to a large extent (Holroyd and McClure, 2015; Laubach et al., 2015; Verguts et al., 2015; Kolling et al., 2016). The human fMRI study reported that the posterior cingulate cortex (PCC) is associated with prospective memories (Andrews-Hanna et al., 2010). Silverman et al. (2015) discovered that PCC involved in reward processing in adolescent. The anterior cingulate may take part in the integration of emotional and control mechanisms (Pessoa, 2009). The ACC could influence response monitoring (Taylor et al., 2007).

The abnormal cingulate gyrus was found in several ASD studies. For example, Luna et al. (2002) discovered that subjects with ASD showed less task-related activation in PCC and dorsolateral prefrontal cortex when executing the space working memory task rather than (HC). Philip et al. (2012) discovered more activation of cingulate gyrus in controls compared to ASD patients. Doyle-Thomas et al. (2012) discovered that there is increased surface area (SA) of the right cingulate cortex in ASD patients. There is abnormal activation of ACC in ASD when performing tasks with different cognitive (Ashwin et al., 2007; Dichter and Belger, 2007). Kana et al. (2007) found abnormal connectivity between ACC and other regions in ASD during a requiring response task.

The cingulate gyrus abnormalities can be seen as the mark of ASD and which probably cause cognitive processes impairment and response monitoring disorder in ASD patients. The experimental result may enhance the clinical diagnosis and treatment of ASD.

To identity ASD patients from TD, we proposed a novel method, random SVM cluster, which has a better classification performance (accuracy is 96.15%). But it also has few limitations. Firstly, we only employed the brain level features in this paper, and we could employ voxel level features in the future studies. Secondly, our study only used four graph metrics as features. In the future studies, we could use more kinds of graph metrics as features. Finally, the random SVM cluster has excellent performance based on only one modal feature, we could boost its performance by multi-modal feature in the future studies.

## Ethics statement

This study was carried out in accordance with the recommendations of National Institute of Aging-Alzheimer's. This study was carried out in accordance s Association (NIA-AA) workgroup guidelines, IRB. The study was approved by IRB of each participating site, including the Banner Alzheimer's Institute, and was conducted in accordance with Federal Regulations, the Internal Conference on Harmonization (ICH), and Good Clinical Practices (GCP).

## Author contributions

XB proposed the design of the work and revised it critically for important intellectual content. QSun and QX carried out the experiment for the work and drafted part of the work. YW and QShu collected, interpreted the data, and drafted part of the work. All the authors approved the final version to be published and agreed to be accountable for all aspects of the work in ensuring that questions related to the accuracy or integrity of any part of the work are appropriately investigated and resolved.

### Conflict of interest statement

The authors declare that the research was conducted in the absence of any commercial or financial relationships that could be construed as a potential conflict of interest.
